# Effect of Ethanolic Extract of *Indigofera tinctoria* on Chemically-Induced Seizures and Brain GABA Levels in Albino Rats

**Published:** 2011

**Authors:** Asuntha Garbhapu, Prasannaraju Yalavarthi, Prasad Koganti

**Affiliations:** 1 *Department of Pharmacy, SPW Polytechnic, Tirupati – 517 502, India *; 2 *Faculty of Pharmaceutical Sciences, UCSI University, Menara Gading, UCSI Heights, 56000 Kuala Lumpur, Malaysia*; 3 *Institute of Pharmaceutical Technology, Sri Padmavati Mahila Visvavidyalayam (A Women’s University), Tirupati -517 502, India*

**Keywords:** Clonic seizures, GABA, GABA-T, Hind limb tonic extension, Indigofera tinctoria, MES, Neurotoxic, PTZ

## Abstract

**Objective(s):**

*Indigofera tinctoria* Linn. of Fabaceae family is claimed to be useful to control epileptic seizures in the Indian system of folkore medicine. This study was designed to evaluate * tinctoria* and to verify the claim.

**Materials and Methods:**

Seizures were induced in male albino rats with pentylenetetrazole (PTZ). The test group animals were administered ethanolic extract of *Indigofera tinctoria* (EEIT) orally. The time of onset and duration of clonic convulsions were recorded. Maximal electroshock seizures (MES) were induced in animals. The duration of hind limb tonic extension (HLTE) was recorded. GABA levels and GABA transaminase activity in brain were estimated.

**Results:**

In PTZ model, EEIT significantly (*P*< 0.01, *P*< 0.001) delayed the onset of convulsions and reduced the duration of seizures in a dose dependent manner. A significant (*P*< 0.05) reduction in the duration of HLTE at higher doses of EEIT was observed in MES model. Increase in brain GABA levels was observed on treatment with EEIT at 500 and 1000 mg/kg doses, suggested that the plant may be acting by facilitating GABAergic transmission. A significant reduction (*P*< 0.05) in the activity of brain GABA transaminase was observed at higher doses. No neurotoxic signs were observed with rotarod test, pentobarbital induced sleeping time, locomotor activity and haloperidol-induced catalepsy.

**Conclusion:**

The ethanolic extract of * tinctoria* was found to be useful to control and treat the variety of seizures.

## Introduction

Seizures are the most common chronic neurological disorder. Worldwide, the prevalence is estimated to be 0.51% and a lifetime incidence of 13% (1). The etiology of seizures comprises various pathophysiological conditions. Change of gammaminobutyric acid (GABA) levels in the brain is highly correlated with seizure development in humans. Antiepileptic drugs (AED) such as barbiturates, some steroids and benzodiazepines modulate GABA_A_ receptors to open a chloride ion selective channel (2). AED elicit their effect as positive allosteric modulators by increasing channel-opening frequency and confer anxiolytic, anticonvulsant, sedative, hypnotic and muscle-relaxant effects (3). At the same time AED may also cause neuronal damages. Over the past decade, an intensive search has been undertaken to find new, more selective drug candidates with fewer side effects for treatment of seizures and other central nervous system (CNS) disorders. Drugs from plant sources hold promise as anti-seizure agents. Although the actions of many medicinal plants are well known, information on their mechanism of action and their active forms *in vivo* is limited. 

Medicinal herbs have been used in over 5000 years. The medical culture of contains both folk traditions and codified knowledge systems with references in the *Adharva Veda,* being textual evidence of the traditional use of medicinal plants. It is estimated that about 80,000 species of plants are utilized by different systems of Indian medicine. The codified traditions have about 75,000 plant drugs and 50,000 formulations are believed to be folk and tribal traditions. In *Ayurveda* (traditional Indian medicine) about 2,000 plant species are considered to have medicinal value, while the Chinese pharmacopoeia lists over 5,700 traditional medicine, most of which of plant origin. Many of the thousands of plant species growing throughout the world have medicinal uses, containing active constituents that have a direct pharmacological action on the body. Several plants used for the treatment of seizures in different systems of traditional medicine have shown activity when tested on modern bioassays for the detection of anti-seizure activity (4) and many such plants remain to be scientifically investigated. 

*Indigofera tinctoria* is claimed to be an anti-seizure agent according to the Indian indigenous system of medicine and methanolic extract of *I. tinctoria* was showing anti-seizure activity in some experimental models (5). However, no studies have been done till date to demonstrate brain GABA level changes in response to its anti-seizure activity of * tinctoria*. Hence, this present study was carried out to systematically evaluate the effect of * tinctoria* on the brain GABA levels, GABA transaminase (GABA-T) activity, neurotoxicity and seizures. The rationale behind selecting the plant is its extensive use by the practitioners of traditional and folklore medicine in the treatment of epilepsy in .

## Materials and Methods

***Drugs and chemicals***

Pentylenetetrazole, artificial cerebrospinal fluid (ACSF), GABA and sodium pentobarbital were purchased from . Diazepam, phenytoin, vigabatrin, chlorpromazine and haloperidol were obtained from Ranbaxy, . Tween 80, ethanol, methanol, HPLC grade chemicals and solvents of analytical grade were purchased from SD Fine Chem, .

***Ethanolic extract of Indigofera tinctoria (EEIT)***

The whole plant of *I. tinctoria *was collected from Srinivasa Mangapuram area, Tirupati, Chittoor district, in the month of June–July matching its seasonal availability. The plant was identified and authenticated by Prof. T Vedavathi (Retd.), Department of Botany, Sri Venkateswara Arts and , . A voucher specimen was deposited at the herbarium, Institute of Pharmaceutical Technology, A Women’s University, Tirupati, India (ref. no.: 02/IT/08-IPT-SPMVV/TPT/2004). The collected plant material was shade dried at room temperature in the laboratory. After drying, the plant material was powdered and passed through the sieve no. 355. About 200 g of plant powder was extracted with 1000 ml of 95% ethanol under reflux by heating over a water bath. The extracts were then vacuum dried. The yield of ethanolic extract of * tinctoria* was 28.22% (w/w). Suspensions of plant extract were prepared with 2% Tween 80, before administration to animals.

***Experimental animals***

Male albino rats of Wistar strain weighing 150-200 g were used for the study. They were housed in polypropylene cages and maintained under standard laboratory conditions with a 12-12 hr light–dark cycle and as well as free access to standard rat pellet diet (Lipton India Ltd.) and drinking water. They were acclimatized to laboratory conditions for 10 days before starting the experiment. The experimental protocol was approved by the institutional animal ethical committee (ref. no: 1220/a/08/CPCSEA).

***Acute toxicity and gross behavioral changes***

The extract used in this study was subjected to acute toxicity studies. The rats were fasted overnight with free access to drinking water and divided into six groups, each containing six animals. Group 1 animals served as control and received distilled water orally (2 ml/kg). Animals in Groups 2 to 6 received 0.25, 0.5, 1.0, 2.0 and 4.0 g/kg, respectively, of the extract orally by gastric intubation using a soft rubber catheter. The animals were observed continuously for 2 hr and then intermittently at one-hourly interval until 24th hr, their behavioural, neurological and autonomic profiles were noted. The animals were observed for mortality up to the 48th hr.

***Assessment of antiepileptic activity***

***Maximal Electroshock (MES) test***

Maximal electroshock seizures were induced by an electroconvulsiometer (INCO, ). The electrical stimulus (150 mA, 0.2 sec duration) was applied through ear electrodes (6). On giving the shock in normal animals, it should have a profile of tonic flexion, extension followed by clonus. Animals were selected by giving the shock 24 hr before the day of experiment. Animals that had shown all the three phases of convulsions were selected for the study. 

Thirty animals were randomly divided into five groups (n= 6). Group 1 animals were kept as control and were administered distilled water (2.0 ml/kg, oral). Group 2 animals were treated with phenytoin (25 mg/kg, oral) as a reference standard. Group 3–5 animals were administered 250, 500 and 1000 mg/kg of the EEIT orally. One hour after administration of the extract, maximal electroshock seizures were induced by an electroconvulsiometer (INCO, ). The electrical stimulus (150 mA, 0.2 sec duration) was applied through ear electrodes. Duration of hind limb tonic extension (HLTE) was recorded. The abolition or reduction in the duration of tonic extension was considered as the index for antiepileptic activity. Percentage protection against seizures was calculated by considering the duration of tonic hind limb extension in reference standard group as 100% (7,8).

***Pentylenetetrazole (PTZ) induced seizure test***

In this model, the animals were randomly divided into 5 groups (n= 6). Group 1 animals were kept as the control and were administered distilled water (2.0 ml/kg, oral). Group 2 animals were treated with diazepam (2.0 mg/kg, i.p.) as a reference standard. Group 3–5 animals were administered 250, 500 and 1000 mg/kg of EEIT orally. One hour after treatment, PTZ (70 mg/kg, i.p.) was injected (9,10). The time of onset and duration of clonic convulsions were recorded. Percentage protection against seizures was calculated by considering the duration of seizures in reference standard group as 100% (11,12).

***Estimation of GABA levels in the brain***

In the present work, GABA levels in the brain homogenate were estimated by HPLC (13) and the studies were carried out on a Hitachi 655A liquid chromatograph connected to a variable wavelength UV monitor. A Rheodyne 7125 injector and 2500 chromato-injector (Hitachi Pvt. Ltd, , ), Phenomenex C18, 5 m (1504.6 mm I.D) () were used.

***Preparation of the brain tissue homogenate***

Five groups were used (n= 6). Group 1 animals (control) were administered distilled water (2.0 ml/kg, oral) while group 2 animals were administered diazepam (2 mg/kg, i.p.) as the reference standard drug for GABA levels estimation, vigabatrin (50 mg/kg, i.p.) was used as reference standard drug for the GABA-T studies. Groups 3-5 animals were administered 250, 500 and 1000 mg/kg of EEIT orally. One hour after administration of the extract, PTZ (70 mg/kg) was injected subcutaneously to all the animals in groups 2-5 animals. On observing onset of convulsions following the administration of PTZ, the animals (including control group) were sacrificed by decapitation. The brains were removed and immediately submerged in ice-cold artificial cerebrospinal fluid (ACSF). The brain tissues were then washed to remove blood, blotted to dry and submerged in 5 ml of methanol, homogenized using a glass Teflon homogenizer for 2 min and centrifuged at 10,000 rpm at 10 °C for 15 min (14).

***Determination of GABA from the brain homogenate***

For GABA quantification, 1 ml from each of the supernatant of brain homogenate and methanol were mixed together and centrifuged at 12000 rpm for 10 min. To a volumetric flask, 0.7 ml supernatant and 0.6 ml of borax buffer of pH8 were added, heated on a water bath at 80 C for 10 min and the final volume was adjusted to 5 ml with methanol. The 5 l solution was injected on phenomenex C18 and eluted with methanol: water (62:38 v/v) with a flow-rate of 1 ml/min. The concentrations were observed with the UV detector at 330 nm (13). 

***Determination of GABA transaminase (GABA-T) activity in the brain***

GABA-T activity in the brain was measured spectrophotometrically (14). To a 10 ml volumetric flask, 15 µmol from each of α-oxoglutarate and GABA, 10 µg of pyridoxal phosphate and 1 ml of supernatant of the brain tissue homogenate were added and the final volume was made up to 3 ml with buffer containing 0.2 M Tris-HCl (pH 8.6). The final mixture was incubated at 37 °C for 30 min for reaction. The reaction was terminated by the addition of 0.5 ml ice-cold 20% trichloroacetic acid. The blank was prepared by replacing the homogenate with methanol from the mixture. The succinic semialdehyde (SSA) produced in the incubation mixture was estimated at 610 nm. The colour complex of SSA and 3-methyl-2-benzothia-zolone-2-hydrazone in the presence of 12% FeCl_3 _was measured against the blank. GABA-T activity was measured in units/mg of protein. 

***Assessment of neurotoxicity***

***Rotarod test***

Sedation, decreased locomotor activity and ataxia were quantified by the rotarod test. Skeletal muscle relaxation induced by a test compound could be evaluated by testing the ability of rats to remain on a rotating rod (15).

The animals were divided into 4 groups (n= 6) and group 1 animals were kept as the control and were administered distilled water (2.0 ml/kg, oral). Group 2–4 animals were administered 250, 500 and 1000 mg/kg of EEIT orally. One hour after administration, the animals were placed on rotarod (INCO, Ambala) and the time each animal could walk continuously on the rotarod and the number of falls from the rotarod during 120 sec were recorded. 

***Pentobarbital-induced sleeping time***

The animals were randomly divided into 5 groups (n= 6). Group 1 animals were kept as the control and were administered distilled water (2.0 ml/kg, oral). Group 2 animals were treated with diazepam (2 mg/kg, i.p.). Group 3–5 animals were administered 250, 500 and 1000 mg/kg of EEIT orally. Sixty minutes later, pentobarbital sodium (30 mg/kg, i.p.) was administered to each animal to induce sleep. Each animal was placed gently on its back after the treatment with pentobarbital. If the animal remained on its back for 30 sec, loss of the righting reflex was considered to occur (15). Animals were observed for the onset and duration of sleep. The duration of sleep was measured as the interval between the loss and the recovery of the righting reflex. 

***Locomotor activity***

The locomotor activity can be an index of wakefulness (alertness) of mental activity. Spontaneous motor activity was monitored using actophotometer (INCO, Ambala), operates on photoelectric cells, which are connected to circuit with a counter. The interceptions made by the animal were counted for a period of 10 mins for each animal. The animals were divided into 5 groups (n= 6) and group 1 animals were kept as the control and were administered distilled water (2.0 ml/kg, oral). Group 2 animals were treated with chlorpromazine HCl (3 mg/kg, i.p.) as reference standard. Group 3-5 animals were administered 250, 500 and 1000 mg/kg of EEIT orally. All animals were tested for locomotor activity. Increase in count was regarded as central nervous stimulation, decrease in count as central nervous depressant activity (15). 

***Haloperidol-induced catalepsy***

Catalepsy, the acceptance and retention of abnormal posture is measured by means of bar test. Antipsychotic or neuroleptic drugs, which block central dopamine receptors, produce a behavioral state in animals in which they fail to correct externally imposed posture (15). The animals were divided into 4 groups (n= 6). Group 1 animals were kept as the control and were administered distilled water (2.0 ml/kg, oral). Group 2-4 animals were administered 250, 500 and 1000 mg/kg of EEIT orally one hour before haloperidol (2 mg/kg/i.p.) administration. The animals were observed for duration of catalepsy at 0, 30, 60 and 90 min after haloperidol administration. The time the animal maintained on imposed position with both fore paws extended on a wooden bar, placed horizontally at a height of 10 cm was recorded. 

***Statistical analysis***

The data was expressed as mean±SEM. ANOVA was used for multiple comparisons followed by Tukey–Krammer test. *P*< 0.05 was considered statistically significant.

## Results

***Acute toxicity and gross behavioral changes***

The extract of *I. tinctoria* did not provoke any gross behavioral changes or manifestations of toxic symptoms in the animals such as weight loss, increased motor activity, tremors, clonic convulsions, tonic extensions, muscle spasm, spasticity, loss of right reflex, decreased motor activity, ataxia, sedation, muscle relaxation, hypnosis, arching and rolling, lacrimation, salivation, viscid, watery diarrhea, writhing and urination over a period of 24 hr. Ethanolic extract of *I. tinctoria* was non-lethal even at the maximum single oral dose of 4.0 g/kg. Hence, the oral doses of the extracts selected for the study were 250, 500 and 1000 mg/kg.

***Effect on MES-induced tonic seizures***

EEIT exhibited significant (p<0.05) reduction in the duration of HLTE at doses of 500 and 1000 mg/kg, orally. The animals were protected 30.13% against seizures induced by MES at dose of 1000 mg/kg, orally when compared to the control group. The results are depicted inFigure 1. 

**Figure 1.**
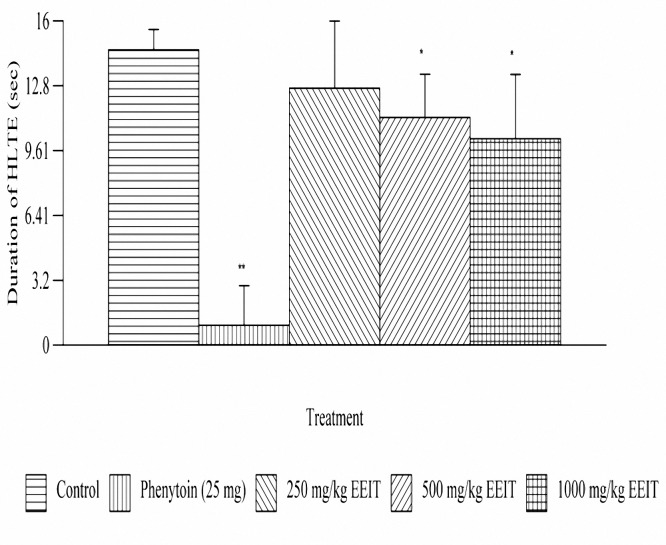
Effect of ethanolic extract of *Indigofera tinctoria* on MES induced tonic seizures of male albino rats of Wistar strain. *P< 0.05, ** *P*< 0.001 as compared to the control

***Effect on PTZ induced seizures***

Treatment with EEIT, significantly (*P*< 0.01, *P*< 0.001) delayed the onset of convulsions in a dose dependent manner. The duration of convulsions was also significantly reduced. The extract has shown a percentage protection of 66.79, 74.45 and 82 at doses 250, 500 and 1000 mg/kg, respectively. The results are depicted in Figures 2A and 2B.

***Effect on the brain GABA levels***

In the EEIT treated animal groups, a significant (*P*< 0.01) increase in the brain GABA levels at 500 and 1000 mg/kg, oral, doses was observed. The percentages of increase in GABA levels were 34.93 and 89.98% at the above tested doses compared to the control group. The results are depicted in Figure 3. 

***Effect on the brain GABA-T activity***

The animals treated with EEIT, showed a significant (*P*< 0.05) reduction in the brain GABA-T activity at 500 and 1000 mg/kg, oral, doses. The percentages of inhibition of GABA-T activity were 59.64 and 64.23% respectively compared to the control group. The results are depicted in Figure 4. 

***Effect on rotarod test***

EEIT did not interfere with motor coordination and did not exhibit difference from vehicle treated animals and all the animals demonstrated no loss of muscle relaxation for more than 120 sec on the rotarod.

***Effect on pentobarbital-induced sleeping time***

The ethanolic extract of * tincoria *showed a slight increase in duration of sleeping time induced by pentobarbitone, but it was not significant as compared to the control group. 

***Effect on locomotor activity***

The parameter assessed was the number of interceptions made by the animal in this model. EEIT did not show significant increase in the number of interceptions. But the animals exhibited slight decrease in the number of interceptions and that extent of decrease was not significant as compared to the control group.

***Effect on haloperidol induced catalepsy***

Duration of catalepsy was the parameter observed in this model. The control group animals exhibited the duration of catalepsy of 297.31±26.33, 297.50±31.22 and 299.66±38.33 at 30, 60, and 90 min respectively. Animals treated with EEIT*, *did not increase the severity of haloperidol induced catalepsy.

**Figure 2A.**
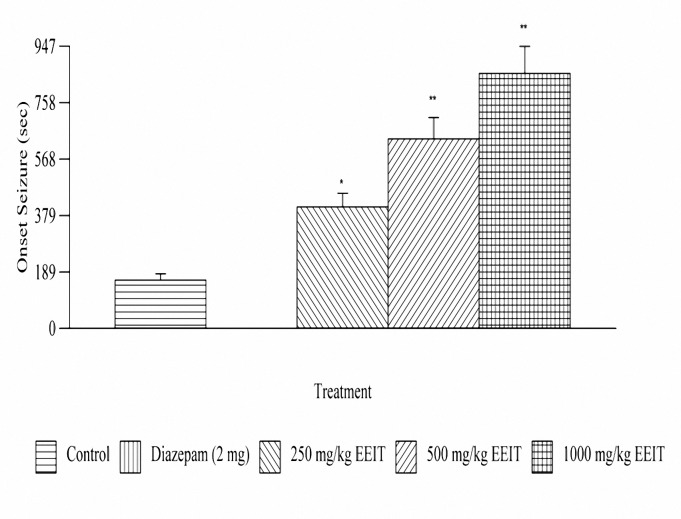
Effect of ethanolic extract of *Indigofera tinctoria* on PTZ induced onset seizures of male albino rats of Wistar strain. *P< 0.01, ** P< 0.001 as compared to the control

**Figure 2B.**
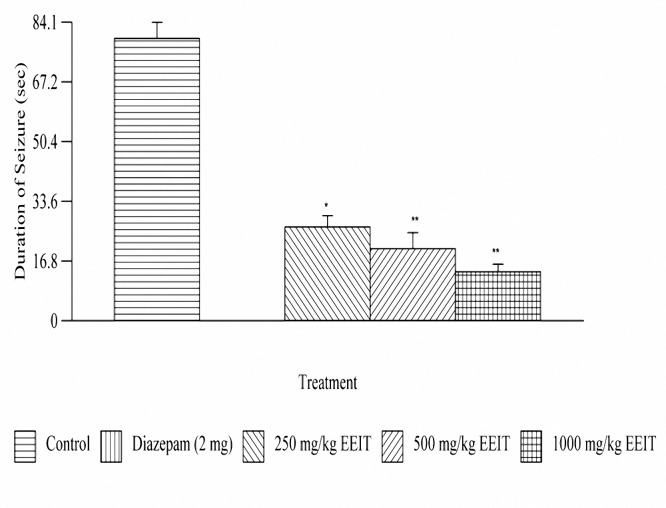
Effect of ethanolic extract of *Indigofera tinctoria* on PTZ induced seizure duration of male albino rats of Wistar strain. * P< 0.01, **P< 0.001 as compared to control

**Figure 3.**
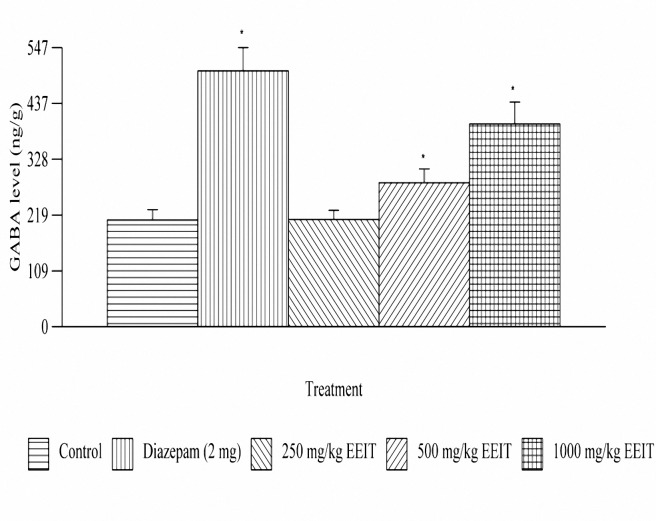
Effect of ethanolic extract of *Indigofera tinctoria* on brain GABA levels of male albino rats of Wistar strain. *P< 0.01 as compared to the control

**Figure 4.**
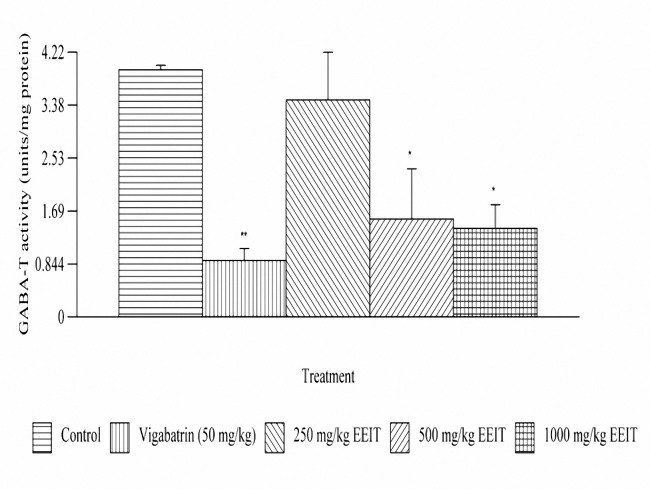
Effect of ethanolic extract of *Indigofera tinctoria* on brain GABA-T activity of male albino rats of Wistar strain. *P< 0.05, **P< .01 as compared to the control

## Discussion

Epileptic seizures are common problems of medical practice. The great majority of studies of the prevalence of epilepsy have reported rates between 4 and 10 per 1000 (16,17). The management of most epilepsies is fairly satisfactory with the range of drugs available. However, a certain percentage of epilepsies do not respond to even polypharmaceutical therapy termed refractory epilepsy, and the risk of toxicity is very high with such therapy (18). Also, the available range of drugs for use in epilepsy itself indicates that there is no single satisfactory drug to meet the needs of therapy (19, 20). Hence the necessity exists for exploring newer drugs, for either greater anti- seizure activity, or lesser side effects or lesser interaction when given in combination with other drugs. Botanical sources remain a huge untapped resource for exploring for such new agents, hence this trial was initiated. Seizures are manifestation of a variety of diseases with variable mortality. Deaths could be due to the causative etiology itself, such as tumors, degenerative conditions or cerebrovascular diseases (21). Evidence indicates that the imbalance between excitatory and inhibitory neurotransmission in the brain is the main cause for seizure development in both experimental and clinical states (22). 

Experimental models of epileptiform seizures have constituted the nearest physiological approach to the development of new AED to control seizures in epileptic patients. The MES and PTZ induced seizure tests are the primary assays in the conventionally accepted anticonvulsant screening procedure (23). The effectiveness in MES test correlates with efficacy in suppressing generalized tonic–clonic and partial seizures (12). 

Na^+^, Ca^2+^ and K^+^ channels regulates the epileptic form discharge, membrane excitability, subthreshold electrical excitability and neurotransmitter release (24). AED have been reported to inhibit voltage gated ion channels, including Na^+^, Ca^2+^ and K^+^ channels (25). MES induced tonic extension can be prevented by AED that inhibit either voltage gated channels or by blocking glutamergic excitation mediated by the N-methyl-D-aspartame receptor (26). AED have the ability to open K^+^ channels in neuronal cells; can prevent seizure induced by PTZ and MES (27).

PTZ, a chemoconvulsant induces seizures by depressing chloride channel function by binding to picrotoxin site on the GABA receptor complex (12). AED that act against PTZ induced convulsions can raise seizure threshold in the brain by enhancing GABA_A_ receptor mediated inhibitory neurotransmission (28). 

Antagonism of PTZ suggests that the EEIT might have effect on GABAergic neurotransmission. Hence, *in vivo* studies were carried on EEIT for their modification of GABA levels in the brain. 

HPLC studies revealed a significant increase (*P*< 0.05) in the brain GABA levels with EEIT as compared to the control group. Our experiment has exhibited comprehensive results to conclude the antiepileptic activity of EEIT. 

GABA is synthesized from glutamate, exclusively in GABAergic neurons, by the action of the enzyme glutamic acid decarboxylase. Upon synaptic release, GABA acts on its three specific receptors, GABA_A_, GABA_B_, and the newly characterized GABA_C_. GABA is removed from the synaptic cleft into localized nerve terminals and glial cells, by specific membrane-bound transport molecules. After removal from the synapse, GABA is either recycled to the readily releasable neurotransmitter pool or metabolized to the inactive molecule succinic acid semialdehyde by the action of the mitochondrial enzyme GABA-transaminase (29). Hence, this study was further extended to find out whether EEIT could reduce the degradation of GABA by +blocking GABA-T. Results of the present study indicate that EEIT reduced the activity of brain GABA-T. 

The term ‘neurotoxic effect’ was used broadly to include the entire gamut of manifestations of nervous system function, which includes behavioral debilitation. AED show neurotoxic effects such as sedation, hypo or hyper–locomotion, ataxia, abnormal gait, reduced or inhibited righting reflexes and muscle relaxation in laboratory animals (30). The EEIT did not exhibit neurotoxicity as there was no sedation, no synergic action on haloperidol induced sleep, shown normal gait, no changes in righting reflexes and all animals were able to maintain equilibrium on rotating rod for more than 120 sec.

## Conclusion

The results of our study clearly demonstrated that ethanolic extract of * tinctoria* has significant ability to control and treat the variety of seizures. Ethanolic extract of *I. tinctoria* has also influenced on the brain GABA levels and GABA-T activity with no neurotoxic signs. Further studies are required to find out the responsible active principle and modes of action of antiepileptic activity of *I. tinctoria* and to establish safer dose.
